# Worse long-term outcomes in new-onset HFpEF vs HFrEF and HFmrEF: findings from the Stockholm PREFERS study

**DOI:** 10.1093/eschf/xvag105

**Published:** 2026-04-09

**Authors:** Hans Persson, Anton Winderud, Camilla Hage, Carin Corovic Cabrera, Ulrika Löfström, Patrik Lyngå, Taner Arslan, Karin Knudsen Malmqvist, Maria J Eriksson, Bengt Persson, Håkan Wallén, Mattias Ekström, Cecilia Linde

**Affiliations:** Department of Cardiology, Danderyd Hospital, 182 88 Stockholm, Sweden; Department of Clinical Sciences, Karolinska Institutet, Danderyd Hospital, 182 88 Stockholm, Sweden; Department of Cardiology, Danderyd Hospital, 182 88 Stockholm, Sweden; Department of Clinical Sciences, Karolinska Institutet, Danderyd Hospital, 182 88 Stockholm, Sweden; Department of Medicine, Karolinska Institutet, Stockholm, Sweden; Department of Cardiology, Karolinska University Hospital, Stockholm, Sweden; Department of Cardiology, South Hospital, Stockholm, Sweden; Department of Clinical Sciences and Education, Södersjukhuset, Karolinska Institutet, Stockholm, Sweden; Department of Medicine, Karolinska Institutet, Stockholm, Sweden; Department of Cardiology, Capio St.Göran Hospital, Stockholm, Sweden; Department of Cardiology, South Hospital, Stockholm, Sweden; Department of Clinical Sciences and Education, Södersjukhuset, Karolinska Institutet, Stockholm, Sweden; Translational Science & Experimental Medicine, Research and Early Development, Cardiovascular, Renal and Metabolism (CVRM), BioPharmaceuticals R&D, AstraZeneca, Gothenburg, Sweden; Department of Cardiology, Danderyd Hospital, 182 88 Stockholm, Sweden; Department of Clinical Sciences, Karolinska Institutet, Danderyd Hospital, 182 88 Stockholm, Sweden; Department of Clinical Physiology, Karolinska University Hospital, Stockholm, Sweden; Department of Molecular Biology and Medicine and Surgery, Karolinska Institutet, Stockholm, Sweden; Science for Life Laboratory, Department of Cell and Molecular Biology, Uppsala University, Uppsala, Sweden; Department of Medical Biochemistry and Biophysics, Science for Life Laboratory, Karolinska Institutet, Stockholm, Sweden; Department of Cardiology, Danderyd Hospital, 182 88 Stockholm, Sweden; Department of Clinical Sciences, Karolinska Institutet, Danderyd Hospital, 182 88 Stockholm, Sweden; Department of Cardiology, Danderyd Hospital, 182 88 Stockholm, Sweden; Department of Clinical Sciences, Karolinska Institutet, Danderyd Hospital, 182 88 Stockholm, Sweden; Department of Medicine, Karolinska Institutet, Stockholm, Sweden; Department of Cardiology, Karolinska University Hospital, Stockholm, Sweden

**Keywords:** HFpEF, HFmrEF, HFrEF, New onset, Outcome, HF clinics

## Abstract

**Background and Aims:**

Prospective outcome data in new-onset heart failure (HF) by ejection fraction (EF) category treated in HF clinics are limited. The Stockholm PREFERS study compared long-term outcomes in new-onset HF with preserved EF (HFpEF), mildly reduced EF (HFmrEF), and reduced EF (HFrEF).

**Methods and results:**

Between 2015 and 2019, 547 patients were enrolled: HFpEF *n* = 135 (25%), HFmrEF *n* = 61 (11%), and HFrEF *n* = 351 (64%). The mean age was 76, 71, and 67 years for HFpEF, HFmrEF, and HFrEF; the median baseline EF was 55%, 45%, and 30% (all *P* < .001 across groups). The primary outcome was time to cardiovascular (CV) mortality or first HF hospitalization (HFH). Secondary outcomes were all-cause mortality, CV mortality, and HFH. NT-proBNP and EF were reassessed at 1 year. The median follow-up was 3.8 years (IQR 3.0–4.7). The risk for the primary outcome was higher in HFpEF than in HFmrEF/HFrEF (unadjusted HR 2.2; 95% CI 1.5–3.1; *P* < .001; adjusted HR 1.7; 95% CI 1.1–2.9; *P* < .05). Overall event rates were: all-cause mortality 12.8%, CV mortality 9.1%, HFH 12.3%. At 1 year, NT-proBNP decreased in HFmrEF by 42% (*P* < .05) and in HFrEF by 55% (*P* < .001), with EF increases of 2% points (pp) (*P* < .05) and 16 pp (*P* < .001), respectively. In HFpEF, EF decreased by 5 pp (*P* < .001) with no change in NT-proBNP.

**Conclusions:**

In new-onset HF managed in university hospital-based HF clinics, HFpEF had worse long-term outcomes than HFmrEF and HFrEF combined. The findings highlight the severity of HFpEF and the need for more effective treatment strategies.

**Clinical trial:**

NCT03671122.

## Graphical Abstract

**Graphical Abstract:**
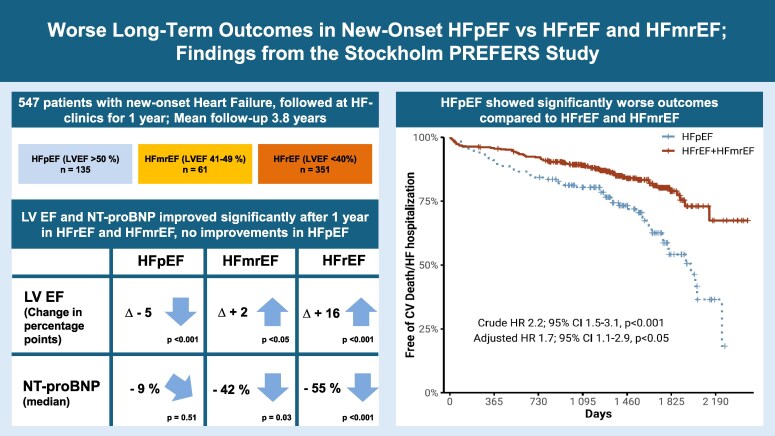

## Introduction

Despite major advances, heart failure (HF) remains common, serious, and associated with high morbidity and mortality. HF with reduced left ventricular ejection fraction (EF) (HFrEF) has established guideline-directed medical therapy (GDMT) that reduces mortality and improves prognosis. In contrast, treatment for HF with preserved EF (HFpEF) is currently limited to sodium-glucose cotransporter-2 inhibitors (SGLT2i) and mineralocorticoid receptor antagonists (MRA), whereas previous recommendations focused only on managing comorbidities and prescribing diuretics.^1,2^ The development of HFpEF therapies has paralleled improvements in diagnostic workup. Since 2021, HFrEF has been defined as left ventricular EF ≤ 40%, HF with mildly reduced EF (HFmrEF) as EF 41%–49%, and HFpEF as EF ≥ 50% combined with HF signs and symptoms and objective evidence of structural or functional abnormalities consistent with elevated left ventricular filling pressures.^2^

Treatment evolution for HFrEF has been highly successful, incorporating four pillars of neurohormonal blockade—betablockers, renin-angiotensin aldosterone system inhibitors (RAASi), MRA, and, more recently, angiotensin receptor-neprilysin inhibitors (ARNI)^3^—along with SGLT2i^4,5^ and device therapy such as cardiac resynchronization therapy (CRT) and implantable cardioverter defibrillators (ICD).^6^ Patients with HFmrEF likely benefit from these therapies, although evidence remains less robust.^1,2^ Although HFpEF has a prognosis comparable to HFrEF,^7,8^ randomized controlled trials (RCTs) for HFpEF were neutral for a long time, only SGLT2i and, more recently, MRA have demonstrated prognostic benefit, primarily by reducing hospitalizations for HF.^9–13^

Importantly, outcomes of new-onset HF have been less studied in RCTs and even less so in prospective registries stratified by HF type (HFrEF, HFmrEF, and HFpEF) and follow-up in HF clinics. The present study was initiated in response to suboptimal quality indicators and poor outcomes reported by the National Swedish Board of Health and Welfare.^14^

### The PREFERS study

The Preserved and Reduced Ejection Fraction Epidemiological Regional Stockholm study (PREFERS) was launched to improve HF management and treatment in Stockholm County, Sweden, in a prospective cohort design within a standardized HF care program at specialized HF clinics.^15^ The primary aim was to compare outcomes in patients with new-onset HFpEF, HFmrEF, and HFrEF. A secondary aim was to explore pathophysiological mechanisms and identify potentially new therapeutic targets for HFpEF, focusing on phenotypes, biomarkers, and proteomics.^16–18^

### Objective

In this prospective study, we compared long-term outcomes among PREFERS patients with new-onset HFpEF, HFmrEF, and HFrEF. We hypothesized a worse outcome for new-onset HFpEF than for HFrEF, primarily due to the lack of GDMT for HFpEF during the study period.

## Methods

Patients with new-onset HFpEF, HFmrEF, and HFrEF were enrolled and followed for 1 year at five university hospital-based HF clinics using the regional 4D standardized HF program. The protocol included visits with HF nurses and HF specialists at study entry and at the one-year follow-up, including analysis of NT-proBNP (ng/L, Roche Diagnostics) and transthoracic Doppler echocardiography performed using a standardized protocol.^16^ Long-term follow-up included all-cause and cardiovascular (CV) mortality, HF hospitalization (HFH), and HF medication use.

### Inclusion and exclusion criteria

The PREFERS study inclusion and exclusion criteria have been previously described.^16^ In brief, patients were eligible if they had signs and symptoms of HF without a previous HF diagnosis, and an elevated NT-proBNP (>300 ng/L for hospitalized patients and >125 ng/L for outpatients). A technically satisfactory echocardiography according to 4D protocol, including Doppler tissue imaging, was required to establish diagnosis of HFpEF, HFmrEF, or HFrEF. The PREFERS echocardiographic protocol and baseline results have been published.^17^ Patients with HFrEF were defined by EF <40%, HFmrEF were defined if EF was ≥40% and <50%,, and HFpEF by EF ≥50% and mean E/e´ > 8.^19^ Exclusion criteria were HF due to valvular disease, hypertrophic obstructive or infiltrative cardiomyopathy, severe chronic obstructive pulmonary disease (COPD), or estimated glomerular filtration (eGFR) ≤ 30 mL/min/1.73 m^2^, or other severe comorbidities that would preclude disable assessment or treatment of HF.

### Ethics

The PREFERS study complies with the Declaration of Helsinki, and the regional appointed ethics committee approved the research protocol; Ethical approval Nr: 2014/846–31/4. Informed consent was obtained from all subjects.

### Outcomes

The prespecified primary outcome was time to CV death or first HFH. Due to the limited number of patients, HFpEF outcomes were primarily compared with the combined group of HFmrEF and HFrEF. Secondary comparisons evaluated HFpEF separately against HFrEF and HFmrEF. NT-proBNP and left ventricular EF by echocardiography were assessed at baseline and after 12 months in all three EF groups. Additional secondary outcomes included the individual components of the primary outcome—CV death, first HFH, and all-cause mortality. Event rates per 100 person-years were calculated.

The additional outcomes EF and NT-proBNP were analysed to reflect HF progression or improvements after 1 year (or last available measurement) in the three HF EF groups. Accordingly, improved HF was defined as a decrease in NT-proBNP or an increase in EF (%), whereas deterioration was defined as an increase in NT-proBNP or a decrease in EF.

### HF clinics and treatment

HF clinics at the five Stockholm university hospitals followed the regional VISS program (2015),^15^ providing guidance on disease management, referrals, and recommended therapies. During the study, evidence-based treatment for HFrEF included RAAS inhibitors, beta-blockers, MRA, diuretics as needed, and CRT/CRT-D when indicated.^6^ ARNIs became available in Sweden in 2017 with slow uptake.^16,17^ For HFpEF, no evidence-based therapy existed; management focused on comorbidities hypertension, diabetes mellitus, ischaemic heart disease, and atrial fibrillation) and to prescribe diuretics as needed. RAASi and MRA were advised for HFpEF with hypertension. Lifestyle and physiotherapy interventions were promoted, and a regional HF physiotherapy program was introduced during the study.

### Statistical methods

Sample size calculations and statistical methods have been previously described.^16,17^ A power calculation including an interim analysis showed that if there was a loss of 20% of the echocardiographic or biomarker data at the 1-year follow up, a total of 400–500 patients would provide sufficient power to assess changes in echocardiographic and biomarker data.^16^ Categorical variables were presented as numbers and percentages, while continuous variables were expressed as means ± standard deviation (SD) or medians with interquartile range (IQR), as appropriate. Group differences were tested using paired or unpaired parametric or non-parametric tests (Wilcoxon, Kruskal-Wallis), as appropriate. Long-term outcomes were analysed using Kaplan–Meier plots.

Unadjusted hazard ratios (HRs) with 95% confidence intervals (CI) were calculated using univariable Cox proportional hazards models. For the primary comparison (HFpEF vs HFmrEF and HFrEF combined), adjusted HRs with 95% CI were computed using multivariable Cox proportional hazards models. The covariates (age, sex, diabetes mellitus, hypertension, eGFR, smoking status, NYHA class, ACE-inhibitor/ARB use, and beta-blocker use) were selected prospectively based on the MAGGIC formula, with the number of primary events (*n* = 117), limiting the number of covariates that could be included in the model.^20^ Sensitivity analyses were performed to assess the impact of additional potential confounders not included in the primary model. Statistical significance was defined as *P* < .05.

## Results

### Patients

Between January 2015 and June 2019, a total of 547 patients, HFpEF *n* = 135 (25%), HFmrEF *n* = 61 (11%), and HFrEF *n* = 351 (64%) were included. The last follow-up of primary outcomes was delayed in some patients due to the COVID-19 pandemic and ended on 31 August 2021. The median follow-up was 3.8 years (IQR 3.0–4.7). Most of the patients (395/547; 72%) were enrolled at the HF outpatient clinics. The remaining 152 patients (28%) were enrolled during or shortly after discharge from their first HF hospital admission, with blood samples collected on average 2–4 weeks later. Key baseline characteristics and Doppler echo-parameters are presented (*Table 1*, *Table 2*, *Table 3*). The mean ages were 76 (HFpEF), 71 (HFmrEF), and 67 (HFrEF) years (*P* < .001). The proportions of female patients were 48% (HFpEF), 30% (HFmrEF), and 31% (HFrEF) (*P* < .001). The median EF was 55%, 45%, and 30% in HFpEF, HFmrEF, and HFrEF, respectively (*P* < .001). HFpEF patients had more comorbidities than HFmrEF and HFrEF patients. Hypertension was the most prevalent comorbidity in HFpEF, 85% vs 70% in HFmrEF, and 54% in HFrEF (*P* < .001). Atrial fibrillation was most prevalent in HFpEF, 59% vs 51% in HFmrEF and 39% in HFrEF (*P* < .001). NYHA class was similar in the three EF groups and with almost all patients in NYHA class II and III.

**Table 1:** Clinical characteristics of HF PREFERS patients categorized according to the proposed universal classification of HF. Continuous variables are presented as medians and lower and upper quartiles (Q1; Q3) and categorical variables as numbers (*n*) and percentages (%)

*Demographics*	HFpEF (LVEF ≥50%) *n* = 135			HFmrEF (LVEF 41–49%) *n* = 61			HFrEF (LVEF ≤40%) *n* = 351			*P*-value overall	*P*-value HFpEF vs. HFrEF
	Median	Q1	Q3	Median	Q1	Q3	Median	Q1	Q3		
Age (years)	76	70	81	71	63	77	67	58	74	<.001	<.001
Sex (females; *n*; %)	65	48		18	30		108	31		<.001	<.001
Body Mass Index (kg/m^2^)	28	25	32	27	24	31	27	24	29	.016	.004
*Previous medical history*	*n*	%		*n*	%		*n*	%			
Atrial fibrillation or flutter	80	59		31	51		137	39		<.001	<.001
Ischaemic heart disease	44	32		23	38		97	28		.243	.345
Hypertension	115	85		43	70		190	54		<.001	<.001
COPD	19	14		4	7		29	8		.121	.045
Diabetes	38	28		13	21		64	18		.073	.022
Renal dysfunction (eGFR <60 ml/kg/min)	45	33		17	28		55	16		<.001	<.001
*Primary HF aetiology*											
Ischaemic	6	4		7	11		36	10		.260	.087
Non-ischaemic	41	31		16	26		91	26			
Dilated cardiomyopathy	1	1		3	5		45	13		<.001	<.001
Hypertrophic cardiomyopathy	2	1		0	0		4	1			
*Clinical findings*											
NYHA class										<.001	.388
I	7	5		14	23		22	6			
II	59	43		29	48		173	50			
III	36	26		12	20		82	23			
IV	0	0		0	0		3	1			
*Treatment* ^a^											
ARB	52	38		18	30		80	23		.004	<.001
ACE inhibitor	42	31		37	61		234	67		<.001	<.001
ARNi	0	0		1	2		6	2		.305	.123
Thiazide HTZ	8	6		1	2		4	1		.009	.003
MRA	32	24		15	25		103	30		.341	.173
Beta blocker	107	79		55	90		320	91		<.001	<.001
Calcium antagonist	45	33		13	21		36	10		<.001	<.001
Furosemide	92	68		33	54		209	60		.120	.091
Antiplatelet	25	18		16	26		99	28		.071	.021
Anticoagulant	32	24		8	13		41	12		.005	.001
ICD	1	1		1	2		6	2		.711	.410
CRT-D	0	0		0	0		2	1		.566	.375

ACE inhibitor, angiotensin-converting enzyme inhibitor; ARB, angiotensin II receptor blocker; ARNi, Angiotensin receptor-neprilysin inhibitor; COPD, chronic obstructive pulmonary disease; CRT-D, cardiac resynchronization therapy-defibrillator; eGFR, estimated glomerular filtration rate; HTZ, hydrochlorothiazide; ICD, implantable cardioverter-defibrillator; MRA, mineralocorticoid receptor antagonists NYHA, New York Heart Association.

^a^May have been instituted at incident heart failure hospitalization estimated to be within previous 4–6 weeks.

**Table 2:** Laboratory and ECG variables in HF PREFERS patients categorized according to the proposed universal classification of HF. Continuous variables are presented as medians and lower and upper quartiles (Q1; Q3) and categorical variables as numbers (*n*) and percentages (%)

Laboratory variable	HFpEF (LVEF ≥50%) n = 135			HFmrEF (LVEF 41–49%) n = 61			HFrEF (LVEF ≤40%) n = 351			P-value overall	P-value HFpEF vs. HFrEF
	Median	Q1	Q3	Median	Q1	Q3	Median	Q1	Q3		
Creatinine (µmol/L)	89	77	108	90	77	104	87	77	100	.437	.288
eGFR (ml/min/1.73m^2^)	68	54	78	71	59	86	74	65	85	<.001	<.001
NT-proBNP (ng/L)	896	462	1645	746	252	1420	1160	563	2370	<.001	.005
hsTroponin T (nanog/L)	16	11	25	17	9	23	14	9	23	.266	.105
Potassium (mmol/L)	4.2	3.9	4.4	4.2	4.0	4.4	4.2	4	4.5	.644	.348
Sodium (mmol/L)	140	139	142	141	140	142	141	139	142	.051	.020
hsCRP (ml/L)	2.3	1.2	4.2	2.2	1.1	4.8	1.6	.77	3.6	.057	.027
Haemoglobin (g/dl)	133	125	142	138	132	148	143	132	154	<.001	<.001
Glucose (mmol/L)	6.1	5.4	7.0	5.9	5.6	6.8	6.0	5.5	6.8	.938	.773
TSH (mE/L)	2.3	1.4	3.2	2.3	1.4	2.8	2.0	1.3	4.0	.978	.870
*Lab 12-months ECG*	*n*	%		*n*	%		*n*	%			
Sinus rhythm	59	52		27	61		194	64		.074	.023
Atrial fibrillation/flutter	51	43		15	34		87	29		.020	.005
	Median	Q1	Q3	Median	Q1	Q3	Median	Q1	Q3		
QRS width (ms)	96	88	118	102	90	136	106	94	140	<.001	<.001

ALT, Alaninaminotransferas; AST, Aspartataminotransferas; eGFR, Estimated Glomerular Filtration Rate (according to the Modification of Diet in Renal Disease study); HbA1c, Hemoglobin A1c; hsCRP, high-sensitivity C-reactive protein; LDL, Low Density Lipoprotein; NT-proBNP, *N*-terminal pro-brain natriuretic peptide; TSH, Thyroid-stimulating hormone.

**Table 3:** Imaging measures in HF PREFERS patients categorized according to the proposed universal classification of HF. Continuous variables are presented as medians and lower and upper quartiles (Q1; Q3) and categorical variables as numbers (*n*) and percentages (%)

Echocardiography	HFpEF (LVEF ≥50%) n = 135			HFmrEF (LVEF 41–49%) n = 61			HFrEF (LVEF ≤40%) n = 351			P-value overall	P-value HFpEF vs. HFrEF
	Median	Q1	Q3	Median	Q1	Q3	Median	Q1	Q3		
LVEDD, (mm)	48	43	52	55	48	61	59	54	64	<.001	<.001
Relative wall thickness	.43	.37	.49	.35	.32	.41	.32	.28	.37	<.001	<.001
LV mass index (g/m^2^)	96	79	116	115	88	139	118	99	143	<.001	<.001
LVH, (*n*; %)	43	31		35	57		179	51		<.001	<.001
LV ejection fraction, (%)	55	53	60	45	43	46	30	25	35	<.001	<.001
LA volume index, (mL/m^2^)	41	31	48	38	30	44	43	34	54	.002	.015
LA volume index >34 (ml/m^2^), (*n*; %)	81	65		39	66		222	75		.085	.044
E/e´ ratio	12.0	9.5	15.0	10.5	8.3	13.7	13.0	10.1	17.2	<.001	.034

LVEDD, Left Ventricular End-Diastolic Diameter; LVH, left ventricular hypertrophy; LA, left atrial; E/e’ ratio, a ratio of mitral E to mean value of septal and lateral tissue e’ velocity.

After inclusion, each patient had an average of three visits to the HF clinic (including nurse visits and a cardiologist visit at the 1-year follow-up), excluding telephone calls and physiotherapy visits.

### Outcomes

**Figure 1:**
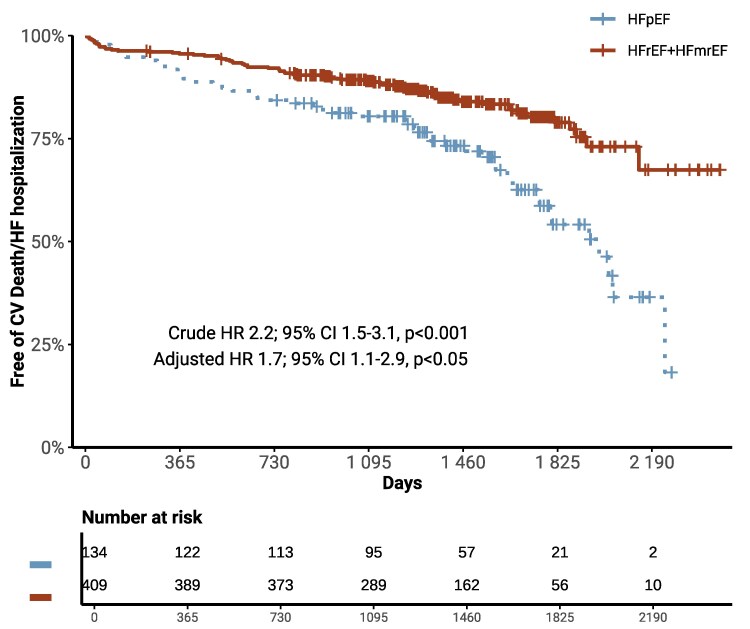
Kaplan–Meier Curves for time to CV death and HF hospitalization in HFpEF vs HFrEF + HFmrEF (*n* = 547).

Over the entire follow-up period of 3.8 years and for the entire cohort, CV mortality/HFH was 21.4% (*n* = 117), all-cause mortality was 12.8% (*n* = 70), CV mortality was 9.1% (*n* = 50), and the first HFH rate was 13.9% (*n* = 67). The combined primary outcome CV mortality/HFH was higher in HFpEF compared with HFmrEF and HFrEF combined (HR 2.2; 95% CI 1.5–3.1, *P* < .001) (*Figure 1*). Adjusted multivariable analysis confirmed an increased risk for HFpEF versus HFmEF and HFrEF (HR 1.7, 95% CI 1.1–2.9, *P* < .05), (*Table 4*).

**Table 4:** Cox regression multivariable analysis comparing HFpEF with HFrEF/HFmrEF, adjusted for nine MAGGIC risk factors, for the primary outcome of CV death or HF hospitalization

Characteristic	HR	95% CI	*P*-value
HF phenotype			
HFrEF and HFmrEF	ref		
HFpEF	1.74	1.06, 2.86	<.05
Sex			
Female	ref		
Male	1.47	.92, 2.36	.11
Age years	1.02	1.00, 1.05	.057
eGFR ml/kg/min	0.98	.96, 0.99	<.001
Hypertension			
No	Ref		
Yes	1.16	0.69, 1.99	.6
Diabetes			
No	ref		
Yes	2.08	1.29, 3.35	<.01
Smoking			
No	ref		
Yes	0.91	0.59, 1.40	.7
NYHA-class			
I	ref		
II or over	9.83	1.36, 71.2	<.05
Treatment with ACEi or ARB			
No	ref		
Yes	1.16	0.68, 1.98	.6
Treatment with betablocker			
No	ref		
Yes	0.62	0.35, 1.11	.11

HFpEF, Heart failure with preserved ejection fraction; HFmrEF, heart failure with mildly reduced ejection fraction; HFrEF, heart failure with reduced ejection fraction; eGFR, Estimated Glomerular Filtration Rate (according to the Modification of Diet in Renal Disease study); NYHA, New York Heart Association; ACEi, angiotensin converting enzyme inhibitor; ARB, angiotensin II receptor blocker.

When analysed separately, the risk for CV mortality/HFH remained higher in HFpEF compared with HFrEF (HR 2.2; 95% CI 1.5–3.1; *P* < .001) and HFmrEF (HR 2.1; 95% CI 1.5–3.1; *P* < .001) (Supplementary Figure). Event rates per 100 patient-years for CV death and HFH were approximately 2–2.5 times higher in HFpEF^10^ compared with HFmrEF (3.9) and HFrEF (4.6), with similarly higher rates for CV death (*Table 5*).

**Table 5:** Event rates per 100 person-years in PREFERS

	HFpEF (LVEF ≥50%) *n* = 135	HFmrEF (LVEF 41–49%) *n* = 61	HFrEF (LVEF ≤40%) *n* = 351
Events CV death	22	5	23
Event rate (%)	4.1	2.4	1.7
Yearly sum	536.4	208	1366.2
Events CV death or HF Hospitalization	49	8	60
Event rate (%)	10.0	3.9	4.6
Yearly sum	491.7	205.8	1295.4

Upper panels: CV Death—Lower panel: CV Death or HF hospitalization (Q1; Q3) and categorical variables as numbers (*n*) and percentages (%). Event rate calculated as follows: Event rate = *N* events/[sum (total years of follow-up for both censored and event individuals)/100] Endpoint: CV death (upper panels); CV Death/HF Hospitalization (lower panels).

### EF and NT-proBNP at baseline and after 1 year

**Figure 2:**
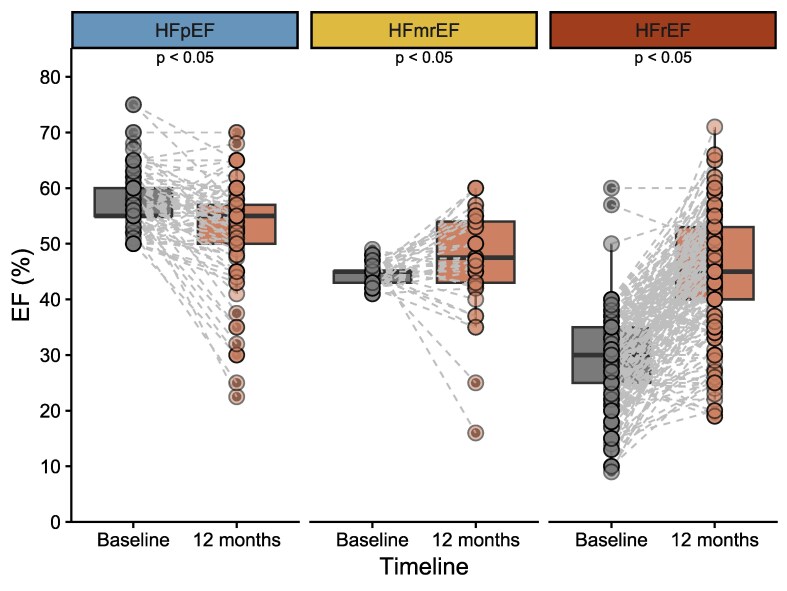
Change in EF from baseline to 12-month follow-up in HFpEF, HFmrEF, and HFrEF.

**Figure 3:**
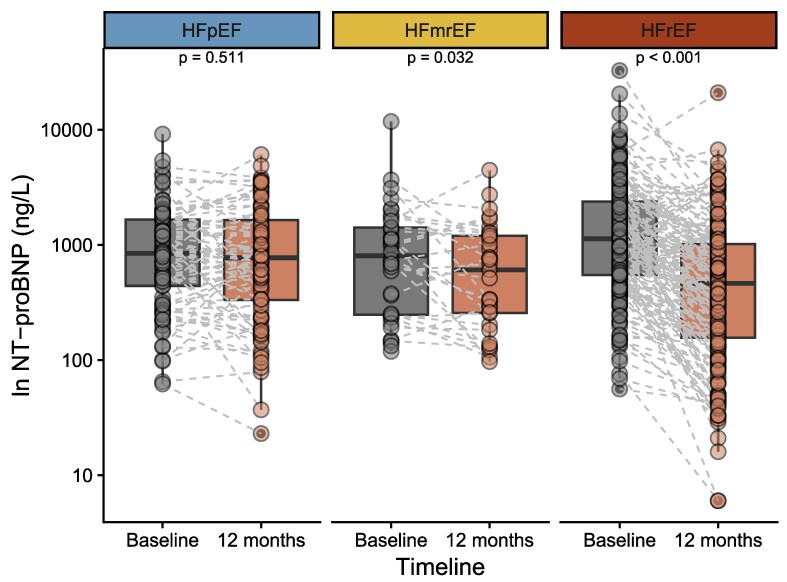
Change in NT-proBNP from baseline to 12-month follow-up in HFpEF, HFmrEF, and HFrEF.

At the 12-month follow-up echocardiography, compared with baseline, mean EF decreased by 5% points (pp) in HFpEF (*P* < .001), increased by 2 pp in HFmrEF (*P* < .05), and increased by 16 pp in HFrEF (*P* < .001) (*Figure 2*, Supplementary Table S1). Median NT-proBNP remained unchanged in HFpEF (−9%, NS) but decreased by 312 ng/L (−42%) in HFmrEF (*P* < .05) and by 538 ng/L (−55%) in HFrEF (*P* < .001) (*Figure 3*, Supplementary Table S2).

A negative correlation was observed between changes in NT-proBNP and EF at the 12-month follow-up in HFmrEF and HFrEF (r = −.22, *P* = .001), indicating lower NT-proBNP with increased EF. Similarly, in HFpEF patients, an increase in EF at 1 year correlated to a decrease in NT-proBNP (r = −.23, *P* = .01).

### Medication and treatments

All EF groups had a high use of ACE-inhibitors, ARBs, or ARNI at baseline, with more extensive use in HFrEF (90%) compared with HFpEF and HFmrEF (70%) (*P* < .001). Few patients in either HFmrEF or HFrEF received ARNI. MRAs were used in similar proportions across all groups, with no significant differences, 24% in HFpEF, 25% in HFmrEF, and 30% in HFrEF (*Table 1*).

**Figure 4:**
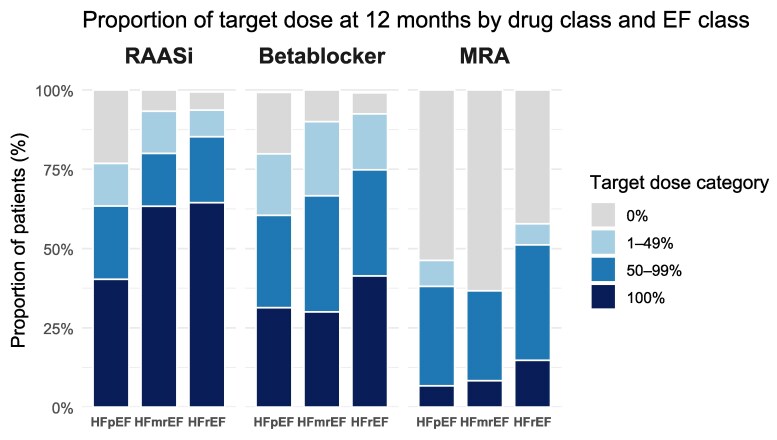
Proportion of target dose of heart failure medication reached at 12 months, stratified by drug class and EF class.

Medication dosages were adjusted during follow-up visits with HF nurses and cardiologists, achieving high or full doses of RAAS inhibitors and beta-blockers in most patients, regardless of EF. At the 12-month follow-up, 51% of the patients had MRAs, including 46% of those with HFpEF. Proportions of patients achieving target doses of HF medications, according to the 2021 ESC guidelines^1^ at the 12-month follow-up, are shown in *Figure 4* and Supplementary Table S3, including device therapy.

Physiotherapy was available at all five hospitals, but with limited access, reaching only 5%–10% of the patients.

## Discussion

In this study, we report outcomes in 547 patients with new-onset HFpEF, HFmrEF, and HFrEF who were referred to five university hospital-based HF clinics in Stockholm, Sweden, between 2015 and 2019 for diagnosis and treatment within the prospective PREFERS study.^17^

First, the primary outcome—CV mortality or HFH—was significantly higher for patients with new-onset HFpEF compared with HFmrEF and HFrEF in adjusted analyses, with a median follow-up of 3.8 years.

Second, patients with HFpEF showed a reduction in EF after 1 year and no meaningful change in NT-proBNP levels. These findings may reflect the limited availability to effective therapies for HFpEF during the study period, although causality cannot be established from our data.

Third, patients with HFrEF demonstrated remarkable improvement at 1 year, with a 16-percentage-point increase in EF and a substantial reduction in NT-proBNP. HFmrEF patients also improved, but to a lesser extent.

### Outcomes

A relatively low event rate was observed in our HFrEF group enrolling patients between 2015 and 2019 (*Table 5*). In contrast, an earlier registry study from the Swedish Heart Failure Registry (SwedeHF), including patients enrolled between 2000 and 2012, reported worse prognosis in HFrEF compared with both HFmrEF and HFpEF. In that study, HFmrEF had a similar prognosis to HFpEF in patients aged ≥80 years and a better prognosis than HFpEF in those <80 years.^21^ However, the SwedeHF study was conducted long before PREFERS and with a shorter follow-up of 2.2 years. Additionally, their primary outcome was all-cause mortality and total CV outcomes (CV and HFH), whereas our primary outcome was CV death or HFH.

Comparison between the two studies is further complicated by differences in care settings: all PREFERS patients were treated at specialized HF clinics, compared with only 37% in SwedeHF, and our cohort consisted exclusively of patients with new-onset HF.

We believe that better use of optimal HF therapy dosages (*Figure 4*), early diagnosis and longer follow-up, and structured patient information, all main instruments of HF clinics, may explain our better results in HFrEF and HFmrEF patients.

How are HF prognosis and diagnosis evolving globally, and what is the prognosis with respect to HF type? Data from the US suggest that HF-related mortality declined from 1999 to 2009, then plateaued for several years, and then began increasing in 2012, continuing through 2021. However, these data did not differentiate between HFpEF, HFmrEF, and HFrEF.^22^ In contrast, recent Swedish population data indicate a steady decline of incident HF-related mortality from 1997 to 2022 without any trends being revealed.^23^ Notably, improvements in survival were smaller in patients with HFpEF compared with HFmrEF and HFrEF, which may reflect the limited effectiveness of HFpEF therapies. Similarly, a Spanish study reported trends for higher mortality and increased HF-related outcomes in HFpEF compared with HFmrEF and HFrEF.^24^

Recently, a national retrospective UK study showed worse 1-year outcomes for new-onset HF diagnosed in hospital versus out of hospital,^25^ with HFpEF patients experiencing the poorest prognosis compared with HFrEF. These findings extend and support our results, where most patients were diagnosed in outpatient care.

Two additional heart failure phenotypes—worsening HF (WHF) and advanced HF (AdvHF)—differ in both management and prognosis.^26^ A key feature of PREFERS is that all patients had new-onset HF, whether enrolled after an acute admission or at their first HF clinic visit, which distinguishes this cohort from studies including worsening or advanced HF.

### Study patients (aetiology and comorbidities)

In this study, we evaluated outcomes following the launch of a new systematic regional care program, which included more than a 3-fold increase in visits to HF clinics compared with previous capacity.^15^ We have previously reported that PREFERS patients had similar characteristics to those in the Swedish Heart Failure Registry (SwedeHF) but were younger and had fewer comorbidities.^17^ Previous myocardial infarction (MI) and ischaemic HF aetiology were more common in SwedeHF, likely because a greater proportion of SwedeHF patients were registered during HFH. Additionally, PREFERS patients consented to participate in a research protocol, which may reflect better disease status and greater health literacy compared with the general HF population. These factors underline that PREFERS patients were all new-onset and earlier in their disease course. Nonetheless, the NYHA class was comparable between PREFERS and SwedeHF, indicating similarities in functional status.

### Medication and physiotherapy

Among comorbidities, HFpEF patients had the highest burden, with hypertension present in 85% compared with 70% in HFmrEF and 54% in HFrEF. This aligns with the FINEARTS-HF trial, which reported 88% hypertension in HFpEF/HFmrEF,^12^ underscoring the central role of hypertension in HFpEF.

HFpEF heterogeneity was evident and has been described in detail previously^17^—patients were older, more often women, and had higher BMI. They had more atrial fibrillation, diabetes, pulmonary, and peripheral artery disease, and fewer had prior coronary revascularization. Renal dysfunction, lower haemoglobin, more frequent anaemia, and higher hs-CRP were also more common. These patterns highlight the multimorbid and heterogeneous nature of HFpEF and emphasize the importance of clinical phenotyping when interpreting outcomes.

Use of RAAS inhibitors was high across all EF groups, but the highest in HFrEF (90% vs. 70% in HFmrEF and HFpEF; *P* < .001). MRAs were used in approximately half of the patients across all EF groups. Few patients with HFmrEF or HFrEF received ARNI, largely due to the slow national introduction following approval in 2015 and restrictions imposed by the national authorities (Medical Products Agency).

Medication dosages were progressively increased during nurse and cardiologist visits at HF clinics, aiming to achieve guideline-directed target doses.^27^ According to a recent SwedeHF study, during the PREFERS recruitment period, 57%–61% of both women and men with HFrEF achieved 50%–100% of target doses for ARNI/RAASi and beta-blockers,^28^ while another 25%–30% received up to 30% of target doses. In the present PREFERS study, target doses were reached in 75%–85% of patients for RAASi at the 1-year follow-up, 63–71% for beta-blockers, and 52%–61% for MRAs, irrespective of EF (*Figure 4*, Supplementary Table S3).

Physiotherapy was available at all five hospitals but was provided to a very limited extent due to restricted reimbursement, despite the 4D HF project’s aim to improve HF care.

Our findings show that patients with new-onset HFrEF and HFmrEF achieved measurable improvements in EF and NT-proBNP, consistent with the availability of effective guideline-directed therapy. Patients with HFpEF, in contrast, had poorer outcomes and showed minimal change in these parameters. These patterns suggest differences in treatment responsiveness across EF phenotypes but do not establish causal relationships.

Recent Swedish data demonstrate that patients in outpatient care with suspected HF and elevated NT-proBNP experience high mortality and morbidity within weeks of presentation, yet often face very long delays before diagnosis.^29^ Together with our findings, this underscores the urgent need to shorten the time to HF diagnosis and initiation of treatment—both in suspected and new-onset HF—whether identified in outpatient or hospital settings and regardless of HF phenotype (HFpEF, HFmrEF, or HFrEF).

### Strengths and limitations

We enrolled fewer new-onset HF patients than expected for several reasons: the long wait for financial resources for implementation of expanded HF clinics: long time to build and communicate the program in the hospitals and to the regional 250 primary care centres. A low inflow of referrals of new-onset HF patients to expanded HF clinics may emanate from limited experience in primary care to use NT-proBNP, which was not allowed to use for cost reasons before our project started.

The differences in outcomes—with better results in HFrEF and HFmrEF compared to HFpEF—could partly be related to differences in clinical characteristics. However, several observations strengthen our interpretation.

First, the corroborative information on NT-proBNP and EF at the 12-month follow-up strongly reinforces that we had effective, evidence-based therapy available for HFrEF and HFmrEF, as reflected by improvements in both parameters. HFpEF showed no significant improvement in either measure, but this does not allow causal interpretation regarding therapy.^30^

Second, outcomes remained worse in HFpEF compared to HFrEF and HFmrEF even after adjustment for nine well-known risk factors. With a lower-than-expected number of events, all possible covariates could not be included in the multivariate model. Together with the observational study design, the effect may be influenced by confounding. However, sensitivity analyses adjusting for the setting of diagnosis, NT-proBNP, and atrial fibrillation did not change the results, indicating that our findings are robust.

Third, since our study period, new guidelines have introduced therapies for all HF phenotypes, including SGLT2 inhibitors and ARNIs. Use of these medications has increased in HFrEF and HFmrEF, and MRAs have been added to recommendations for HFpEF, which may improve future outcomes.^31^

Fourth, although the event rate for the primary outcome in PREFERS may appear low, the rate for HFpEF was comparable to that observed in the placebo group of the recent FINEARTS-HF trial of mostly non-hospitalized HFpEF patients, where finerenone demonstrated modest benefit with slightly lower NT-proBNP compared to placebo.^12,30^

Fifth, a few 12-month visits were delayed due to the COVID-19 pandemic, with some follow-up intervals extending up to 18 months. Additionally, six patients had delayed echocardiography beyond 31 August 2021 but were included in the EF analysis. A sensitivity analysis excluding these patients did not alter the results or their statistical significance.

Sixth, in our cohort, information on the underlying aetiology of HFrEF was missing for many patients, making separate analyses of ischemic versus non-ischemic aetiology difficult to interpret and underpowered.

Last, the exclusion of patients with severe renal dysfunction (eGFR ≤30 mL/min/1.73 m^2^) limits generalizability. Advanced chronic kidney disease is common in HF, especially in HFpEF, and is associated with a worse prognosis. Our cohort may therefore represent a lower-risk population. Including patients with more advanced renal impairment in future studies would improve external validity.

## Conclusions

New-onset HFpEF patients treated at university hospital-based HF clinics for 1 year had worse long-term outcomes than those with HFmrEF or HFrEF.

Our conclusions are strongly supported by the substantial improvements in both EF and NT-proBNP in HFrEF, and a similarly large decrease in NT-proBNP in HFmrEF, in contrast to no improvements in HFpEF. The findings highlight the severity of HFpEF and the need for more effective treatment strategies.

## Supplementary Material

xvag105_Supplementary_Data

## Declarations

### Data Availability

No data were generated or analysed for this manuscript.

### Pre-registered Clinical Trial Number

None supplied.
