# Supplementary material for: Worse long-term outcomes in new-onset HFpEF vs HFrEF and HFmrEF: findings from the Stockholm PREFERS study
Source: ESC Heart Fail. 2026 Apr 9;13(3):xvag105. doi: 10.1093/eschf/xvag105 (PMC13365154; doi:10.1093/eschf/xvag105)
Supplement: xvag105_Supplementary_Data [file xvag105_supplementary_data.zip › Suppl Table 1 EF during one year in HF groups 251217.docx]

**Supplementary Table 1.**

**Ejection fraction (EF) at baseline and at 12-months follow-up, by EF-group**

|  | **HFpEF (LVEF ≥50%) n=110** | | |  | **HFmrEF (LVEF 41-49%) n=45** | | |  | **HFrEF (LVEF ≤40%) n=253** | | |
| --- | --- | --- | --- | --- | --- | --- | --- | --- | --- | --- | --- |
|  | **Baseline** | **12m** | **p-value** |  | **Baseline** | **12 m** | **p-value** |  | **Baseline** | **12 m** | **p-value** |
| EF mean (SD) | 57 (5) | 52 (9) | **0.001** |  | 45 (2) | 47 (9) | **0.0019** |  | 29 (8) | 45 (10) | **0.001** |
| EF Median (Q1-Q3) | 55 (55-60) | 55 (50-57) |  |  | 45 (43-45) | 48 (43-54) |  |  | 30 (25-35) | 45 (40-53) |  |
