# Supplementary material for: Worse long-term outcomes in new-onset HFpEF vs HFrEF and HFmrEF: findings from the Stockholm PREFERS study
Source: ESC Heart Fail. 2026 Apr 9;13(3):xvag105. doi: 10.1093/eschf/xvag105 (PMC13365154; doi:10.1093/eschf/xvag105)
Supplement: xvag105_Supplementary_Data [file xvag105_supplementary_data.zip › Suppl Table 2 NTproBNP during one year 251217.docx]

**Supplementary Table 2.**

**NT-proBNP levels at baseline and at 12-months follow-up, by EF-group**

|  | **HFpEF (LVEF ≥50%) n=84** | | |  | **HFmrEF (LVEF 41-49%) n=28** | | |  | **HFrEF (LVEF ≤40%) n=174** | | |
| --- | --- | --- | --- | --- | --- | --- | --- | --- | --- | --- | --- |
|  | **Baseline** | **12m** | **p-value** |  | **Baseline** | **12 m** | **p-value** |  | **Baseline** | **12 m** | **p-value** |
| NT-proBNP mean (SD) | 1,097 (978) | 1,120 (1,137) | 0.704 |  | 1,059 (920) | 812 (920) | **0.043** |  | 1,971 (3,331) | 917 (1,844) | **0.001** |
| NT-proBNP  median (Q1-Q3) | 817 (335-1,575) | 741 (329-1,420) |  |  | 738 (249-1,520) | 426 (193-1,235) |  |  | 980 (547-2,195) | 442 (158-996) |  |
