# Supplementary material for: Worse long-term outcomes in new-onset HFpEF vs HFrEF and HFmrEF: findings from the Stockholm PREFERS study
Source: ESC Heart Fail. 2026 Apr 9;13(3):xvag105. doi: 10.1093/eschf/xvag105 (PMC13365154; doi:10.1093/eschf/xvag105)
Supplement: xvag105_Supplementary_Data [file xvag105_supplementary_data.zip › Suppl Table 3 Pharm and device therapy at 12m 251217.docx]

**Supplementary Table 3.**

**Pharmacological and device therapy at 12-months follow-up, categorized according to the universal classification of HF.**

|  | **HFpEF (LVEF ≥50%) N = 132** | **HFmrEF (LVEF 41-49%) N = 59** | **HFrEF (LVEF ≤40%) N = 344** |
| --- | --- | --- | --- |
| RAASi - n (%) | 102 (77) | 55 (93) | 324 (94) |
| RAASi - % of target dose | 74.9 | 81.2 | 84.7 |
| Betablocker - n (%) | 108 (82) | 53 (90) | 321 (93) |
| Betablocker - % of target dose | 67.1 | 63.4 | 70.9 |
| MRA - n (%) | 61 (46) | 22 (37) | 199 (58) |
| MRA - % of target dose | 52 | 61.4 | 60.1 |
| SGLT2i - n (%) | 1 (0.8) | 1 (1.7) | 6 (1.7) |
| Statin - n (%) | 60 (45) | 35 (59) | 141 (41) |
| Platelet inhibitor - n (%) | 26 (20) | 19 (32) | 81 (24) |
| Oral anticoagulation - n (%) | 81 (61) | 29 (49) | 144 (42) |
| CRT-D - n (%) | 0 (0) | 0 (0) | 6 (1.7) |
| ICD - n (%) | 0 (0) | 0 (0) | 5 (1.5) |

RAASi = Renin-angiotensin-aldesterone system inhibitors, containing angiotensin converting enzyme inhibitors (ACEi), angiotensin II receptor blockers (ARB) and angiotensin receptor – neprilysin inhibitor (ARNI). MRA = Mineral receptor antagonists. SGLT2i = sodium-glucose cotransporter-2 inhibitors. CRT-D = Cardiac resynchronization therapy with defibrillator. ICD = Implantable cardioverter-defibrillator.
