# Supplementary figures and images for: Worse long-term outcomes in new-onset HFpEF vs HFrEF and HFmrEF: findings from the Stockholm PREFERS study
Source: ESC Heart Fail. 2026 Apr 9;13(3):xvag105. doi: 10.1093/eschf/xvag105 (PMC13365154; doi:10.1093/eschf/xvag105)

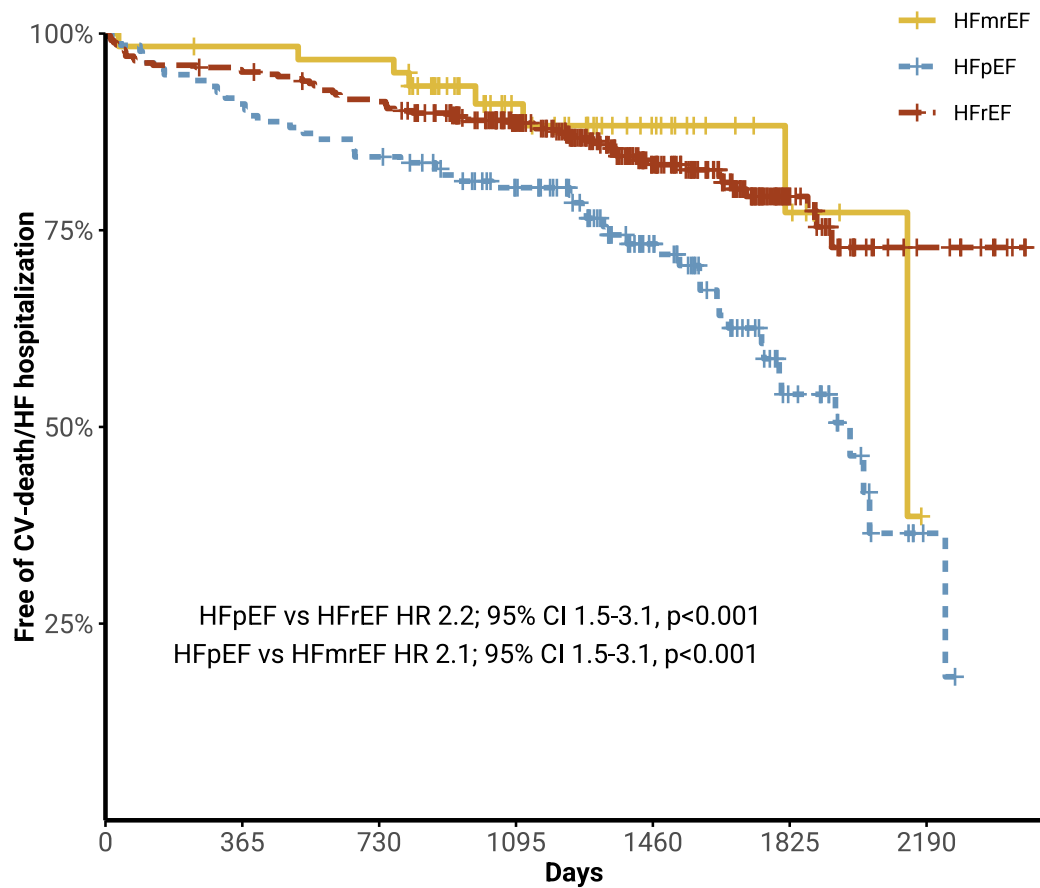

Supplement: xvag105_Supplementary_Data [file xvag105_supplementary_data.zip › Supplementary fig_Surv_Plot_all_LVEF 260318.pdf]
